# Supplementary material for: Cartilage Regeneration Potential in Early Osteoarthritis of the Knee: A Prospective, Randomized, Open, and Blinded Endpoint Study Comparing Adipose-Derived Mesenchymal Stem Cell (ADSC) Therapy Versus Hyaluronic Acid
Source: Int J Mol Sci. 2025 Aug 31;26(17):8476. doi: 10.3390/ijms26178476 (PMC12428590; doi:10.3390/ijms26178476)
Supplement: Supplementary file 1 [file ijms-26-08476-s001.zip › ijms-3823809-supplementary.pdf]

Supplementary data

Cartilage Regeneration Potential in Early Osteoarthritis of the Knee: A Prospective, Randomized, Open, and Blinded Endpoint Study Comparing Adipose-Derived Mesenchymal Stem Cell (ADSC) Therapy Versus Hyaluronic Acid

Ponthep Tangkanjanavelukul [1,*], Saradej Khuangsirikul [2], Danai Heebthamai [2], Montarop Yamabhai [3], Thitima Sumphanapai [4], Nattapat Khumtong [5] and Thanainit Chotanaphuti [2,*,†]

1 Department of Orthopedics, School of Medicine, Institute of Medicine, Suranaree University of Technology, 111 University Avenue, Nakhon Ratchasima 30000, Thailand

2 Department of Orthopaedic, Phramongkutklao College of Medicine, 317 Ratchawithi Road, Thung Phaya Thai, Ratchathewi, Bangkok 10400, Thailand; ksaradej@yahoo.com (S.K.); danaiheeb@hotmail.com (D.H.)

3 Molecular Biotechnology Laboratory, School of Biotechnology, Institute of Agricultural Technology,

Suranaree University of Technology, 111 University Avenue, Nakhon Ratchasima 30000, Thailand; montarop@g.sut.ac.th

4 Division of Clinical Hematology and Microscopy, Department of Medical Technology, School of Allied Health Sciences, University of Phayao, Phayao 56000, Thailand; thitima.su@up.ac.th

5 Department of Family Medicine, School of Medicine, Institute of Medicine, Suranaree University of Technology, 111 University Avenue, Nakhon Ratchasima 30000, Thailand; nattapat.tanwa@gmail.com

* Correspondence: pornthep@g.sut.ac.th (P.T.); thanainit_cho@phyathai.com (T.C.);

† Current Address: Phyathai 2 Hospital, 943 Phahonyothin Rd, Phaya Thai, Bangkok 10400, Thailand.

Supplementary information

Figure S1 Axial and sagittal knee MRI images obtained using the T2-weighted echo spin technique, illustrating before and after intra-articular hyaluronic acid injection. (A) Axial knee MRI images: The arrow shows a stable focal cartilage lesion at the patellofemoral joint, with an unchanged grade 3 focal lesion at the medial patellar facet. (B) Axial knee MRI images: Arrow indicates progressive cartilage degeneration at the mid femoral trachea groove. MRI demonstrates worsening chondral pathology, showing a diffuse grade 3 cartilage lesion at the mid-medial femoral condyle. (C) Sagittal MRI images of the knee: Arrow shows advancing cartilage deterioration in the medial femoral compartment with a diffuse grade 3 cartilage lesion at the mid-medial femoral condyle progressed from grade 2 to 3, accompanied by subjacent chondral bone edema.

Figure S2 The formular for sample size calculation.

Figure S3 The individual conducting the injections was unaware of the specific treatment administered to the patient, whether it was ADSCs or HA.

Table S1. MRI-Based Mean Cartilage Lesion Changes by Location: ADSCs vs. HA Treatment

Figure S1

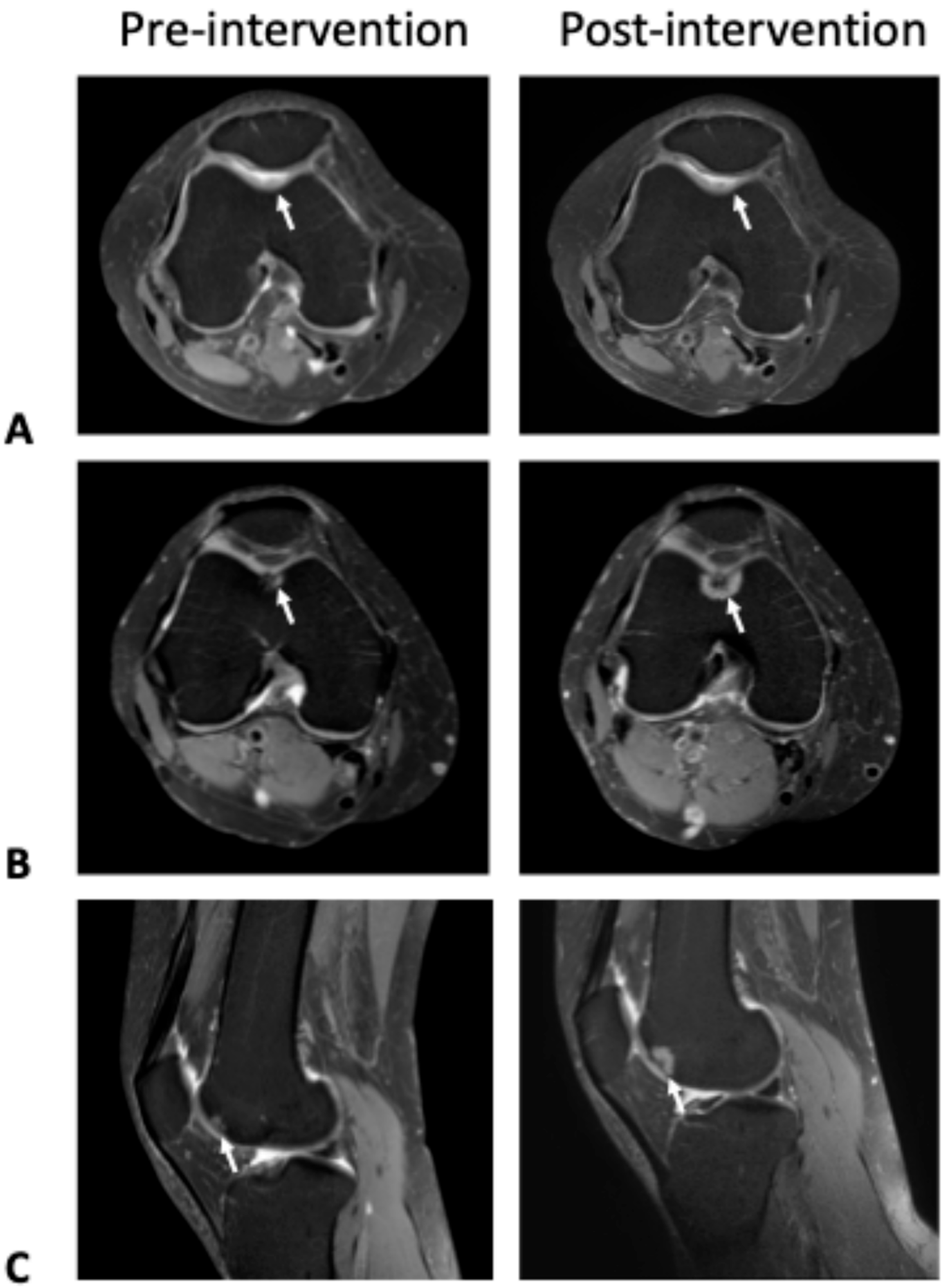

Figure S2

α = 0.05 (one-sided test) $Z_{0.05}$ = 1.64

β = 0.10 $Z_{0.10}$ = 1.28

$$\text{n/group} = \frac{(Z_\alpha + Z_\beta)^2}{(\varepsilon - \delta)^2} \times \left[\frac{p_1(1-p_1)}{k} + p_2(1-p_2)\right]$$

$$= \frac{(1.64+1.28)^2}{(|0.3165-0.2023|-0.5)^2} \times \left[\frac{0.3165(1-0.3165)}{1} + 0.2023(1-0.2023)\right]$$

$$= 21.64$$

$$\approx 22$$

n/group	=	sample size per group
p_1	=	an improvement rate of the Re-Join® group 31.65 %
p_2	=	an improvement rate of the HA group 20.23 %
ε	=	p_1 - p_2
δ	=	Statistical significance of the difference in sizes (Superiority margin) = 0.5
k	=	Ratio between two groups

Figure S3

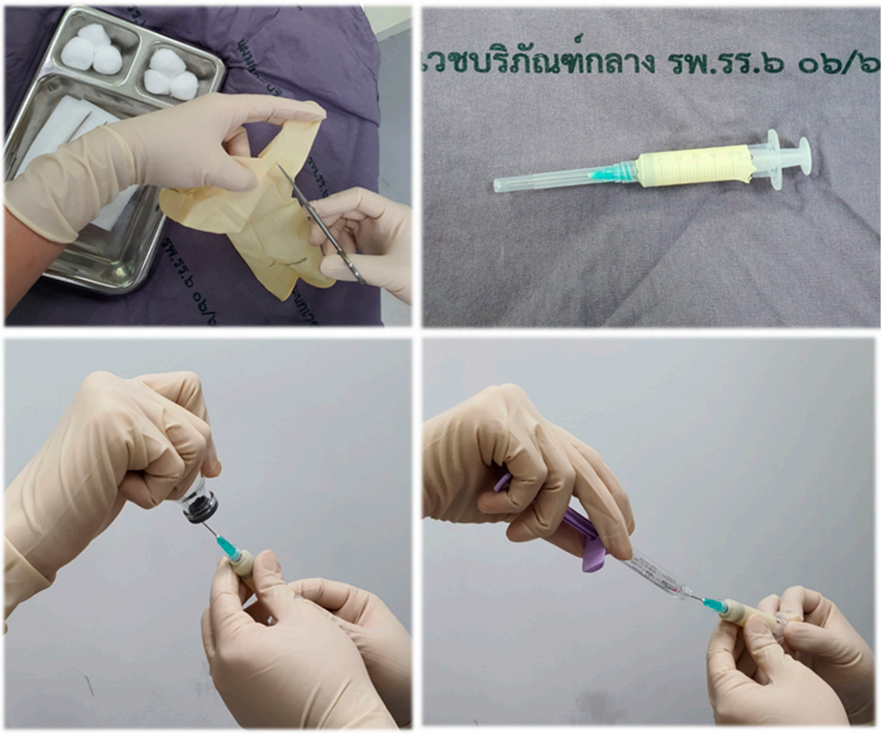

Table S1. MRI-Based Mean Cartilage Lesion Changes by Location: ADSCs vs. HA Treatment

		area change		volume change	
		medial femur (mm^2)	medial patella (mm^2)	medial femur (mm^3)	medial patella (mm^3)
**ADSCs**	mean	-11.78	-9.13	-28.52	-19.78
	95%CI	(-27.42 to 3.85)	(-16.80 to -1.46)	(-65.63 to -8.58)	(-34.25 to -5.31)
**HA**	mean	6.42	3.17	33.38	6.29
	95%CI	(-8.89 to 21.72)	(-4.35 to 10.68)	(-2.95 to 69.70)	(-7.87 to 20.46)
**difference**	mean	-18.2	-12.3	-61.90	-26.07
	95%CI	(-40.08 to 3.68)	(-23.04 to -1.56)	(-113.82 to -9.97)	(-46.32 to -5.83)
***p*-value**[a]		0.103	0.025*	0.019*	0.012*

[a]multilevel mixed effect generalized linear model multilevel mixed effect generalized linear model, *$p < 0.05$
